# Assessing Methods for Assigning SNPs to Genes in Gene-Based Tests of Association Using Common Variants

**DOI:** 10.1371/journal.pone.0062161

**Published:** 2013-05-31

**Authors:** Ashley Petersen, Carolina Alvarez, Scott DeClaire, Nathan L. Tintle

**Affiliations:** 1 Department of Biostatistics, University of Washington, Seattle, Washington, United States of America; 2 Department of Biostatistics, Florida International University, Miami, Florida, United States of America; 3 Department of Mathematics, Hope College, Holland, Michigan, United States of America; 4 Department of Mathematics, Statistics and Computer Science, Dordt College, Sioux Center, Iowa, United States of America; The University of Chicago, United States of America

## Abstract

Gene-based tests of association are frequently applied to common SNPs (MAF>5%) as an alternative to single-marker tests. In this analysis we conduct a variety of simulation studies applied to five popular gene-based tests investigating general trends related to their performance in realistic situations. In particular, we focus on the impact of non-causal SNPs and a variety of LD structures on the behavior of these tests. Ultimately, we find that non-causal SNPs can significantly impact the power of all gene-based tests. On average, we find that the “noise” from 6–12 non-causal SNPs will cancel out the “signal” of one causal SNP across five popular gene-based tests. Furthermore, we find complex and differing behavior of the methods in the presence of LD within and between non-causal and causal SNPs. Ultimately, better approaches for *a priori* prioritization of potentially causal SNPs (e.g., predicting functionality of non-synonymous SNPs), application of these methods to sequenced or fully imputed datasets, and limited use of window-based methods for assigning inter-genic SNPs to genes will improve power. However, significant power loss from non-causal SNPs may remain unless alternative statistical approaches robust to the inclusion of non-causal SNPs are developed.

## Introduction

Increasingly, in the analysis of SNP microarray data, SNPs are aggregated into sets representing genes, pathways, or other biologically meaningful sets. Set-based tests are then conducted in addition to testing for genotype-phenotype association using single marker approaches. The set-based approach is part of a general trend in statistical genetics to leverage *a priori* biological knowledge in the analysis of genetic data, instead of conducting analyses in an agnostic (no prior biological knowledge considered) fashion. However, the traditional, single-marker, agnostic approach toward the analysis of SNP microarray data is by far the most commonly used. In the single-marker approach, researchers typically test each of the 1 million-plus measured or imputed SNPs for evidence of independent association with the phenotype after controlling for relevant covariates. A substantial multiple-testing penalty (e.g., p<1×10^−8^) is then applied to each of the single-marker association test p-values, before deeming a SNP as showing significant evidence of a genotype-phenotype association. With such a small type I error cutoff for statistical significance, designing an adequately powered study can be challenging. Additionally, results to date suggest generally low effect sizes with an average OR of 1.3 in the NHGRI GWAS catalog [1] for most common SNPs (MAF>5%). Thus, in order to have adequate power to find disease associated SNPs, very large sample sizes are needed – especially for SNPs with lower minor allele frequencies, e.g. 5–10%. While designing studies with tens to hundreds of thousands of subjects is possible in some situations, for many diseases it is difficult to obtain a sufficient number of cases.

One promising approach to address these limitations of traditional, agnostic, single marker analyses of SNP microarray data considers testing biologically meaningful sets of SNPs. The two main purposes of this approach are to gain power through (1) aggregating true genotype-phenotype signal across the members of the set and (2) reducing the multiple-testing penalty by reducing the number of tests of significance being conducted. A number of recent approaches for the analysis of common variant SNP data using sets of SNPs have been proposed [2]–[5]. See Petersen, Spratt and Tintle for a more comprehensive listing.[6] Notably, there are similarities between this approach and the approach being proposed for the analysis of rare variants from next-generation sequencing data [7], [8].

While SNP-set methods have frequently been cited in the methodological literature as improving power relative to single-marker tests, in practice, these methods have remained somewhat under-utilized. Much of the literature for SNP-set testing methods applied to common and/or rare variants has considered the question of how to aggregate SNP genotype-phenotype signals statistically and which approaches are most powerful. Less focus has been given to the question of how SNPs should be aggregated into sets. While there are numerous biologically relevant sets (e.g. pathways, genes), we will focus the remainder of our attention on gene-based sets since, to date, this is arguably the most common way to aggregate SNPs. As would be expected, in most situations gene-based tests of association assign all SNPs within a gene (intragenic SNPs) to that gene for the test.

Methods vary, however, when considering inter-genic SNPs (SNPs that exist outside of the start and stop positions of a gene's exonic and intronic regions). The most common approach to assign SNPs to genes is the “Window” approach. In essence, the window approach extends the start and stop positions of the gene an arbitrary amount, ranging from 5 to 500 kb. Typically the window size is the same for both ends of a gene, but it can differ [9]. Rules vary in regards to handling windows that contain other genes. For example, Wang et al. [10] assign each SNP to its nearest gene, as long as that SNP is within 500 kb of the gene. Thus, while SNPs could be assigned to genes up to 500 kb away, the window size is actually much smaller for most genes. Others have used a fixed window approach that assigns all SNPs within 50 kb of the gene. Thus SNPs that are intragenic to other genes, or in intergenic regions not flanked by the gene of interest, could be assigned to a gene [11]. In addition to window-type approaches for assigning SNPs to genes, some authors have only used intragenic SNPs in their analysis [12], [13]. Finally, a few authors have considered assigning intergenic common SNPs to genes if the SNP falls within an LD block spanning the gene (e.g., all SNPs in LD above a certain threshold with SNPs in the gene) [14]. Additionally, others have considered a hybrid approach combining a small window plus intergenic SNPs in LD with SNPs inside of the gene [15].

To date, little work has considered the pros and cons of the various SNP-gene assignment approaches and their potential impact on the performance of gene-based tests. We consider the impact of the inclusion of non-causal SNPs and LD structure on set-based tests of association for common SNP variants using simulated genotype and phenotype data. In each of these scenarios, we consider five SNP-set tests of association, representing two broad classes of tests [6] allowing us to investigate the differential impact of non-causal SNP inclusion and LD structure on these methods. Three tests we consider attempt to combine single-marker p-values into a single set-based p-value, while accounting for LD structure: the GATES method [4] and two different versions of the VEGAS procedure [3]. We also considered two regression based approaches (traditional logistic regression and logistic regression using principal components) that operate on the full genotype-phenotype matrix [5].

## Methods

In order to assess the impact of different methods of assigning SNPs to genes on gene-based tests of association, we simulated genotype and phenotype data. In the following paragraphs we describe the data simulation process. There were four separate genotype simulations conducted as part of this analysis: (1) A simulation of independent genotypes; (2) A simulation of genotypes with LD between non-causal variants only; (3) A simulation of genotypes with LD between causal and non-causal variants; and (4) A realistic genotype simulation involving complex LD structure. In all cases, in order to generate the samples used in the analysis, a large population of genotypes was simulated assuming HWE. Five hundred random case-control samples were generated for each simulation setting.

### Simulation of genotypes with no LD

The simulation with no LD covered 288 separate settings, as follows. Sets of SNP genotypes contained 1, 2, 4, or 8 causal SNPs. The relative risk (defined later) of each SNP set was 1.25 or 2.00, the total sample size (split evenly between cases and controls) was either 2000 or 4000, and the causal SNPMAF was either 5% or 30%. Thus, there were a total of different ways to generate causal SNPs in the set. For each of the 32 causal SNP settings, we considered 9 different non-causal SNP settings (0,2,4,8 or 32 non-causal SNPs at either 5% or 30% MAF), for a total of 288 ( = 9×32) total settings. To simulate a situation with no LD, all SNP genotypes were simulated independently.

### Simulating genotypes with LD between non-causal SNPs

Genotypes were also simulated to include LD structure between the non-causal SNPs. Specifically, non-causal SNP genotypes were recreated for each of the settings described in the previous section, assuming all non-causal SNPs were in the same LD block, or one of two separate LD blocks. LD blocks were in either low (correlation, *r*, of 0.5 between all pairs of SNPs in the block) or high (correlation, *r*, of 0.9 between all pairs of SNPs in the block) correlation. SNP genotypes were generated using the method of simulating correlated binary variables [16].

A total of 896 simulation settings were considered. The settings were closely related to those used for the simulation with no LD. Specifically, the same 32 combinations of parameters for causal SNPs were used, along with the same options for non-causal SNPs with the added component that non-causal SNPs were either all in the same high or low LD block, or non-causal SNPs were evenly split between two separate high, low or high and low LD blocks.

### Simulating genotypes with LD between non-causal and causal SNPs

We also considered LD structure between causal and non-causal SNPs. In this scenario, we assumed each causal SNP was in its own LD block, and that each non-causal SNP was in exactly one LD block with a causal SNP. For each LD block, every non-causal SNP was correlated with the causal SNP to the same degree (either *r* = 0.5 or *r* = 0.9) and all non-causal SNPs within the block had the minimum correlation possible with each other (i.e. *r* = 0.25 for low-LD blocks and *r* = 0.81 for high-LD blocks There were a total of 96 simulation settings, representing all possible combinations of settings above ( = 3 [# of causal SNPs = 1,2,4] ×2 [# of non-causal SNPs = 4,8] ×2 [relative risk = 1.25,2] ×2 [sample size = 2000,4000] ×2 [MAF = 0.05,0.30] ×2 [levels of LD = high, low]). In addition to analyzing all SNPs in the set, we also conducted analyses which ignored all causal SNPs to represent a situation where only tag SNPs were measured, for a total of 192 ( = 2×96) settings analyzed when LD was present between causal and non-causal SNPs.

### Simulation of realistic LD structure

**Figure 1:**
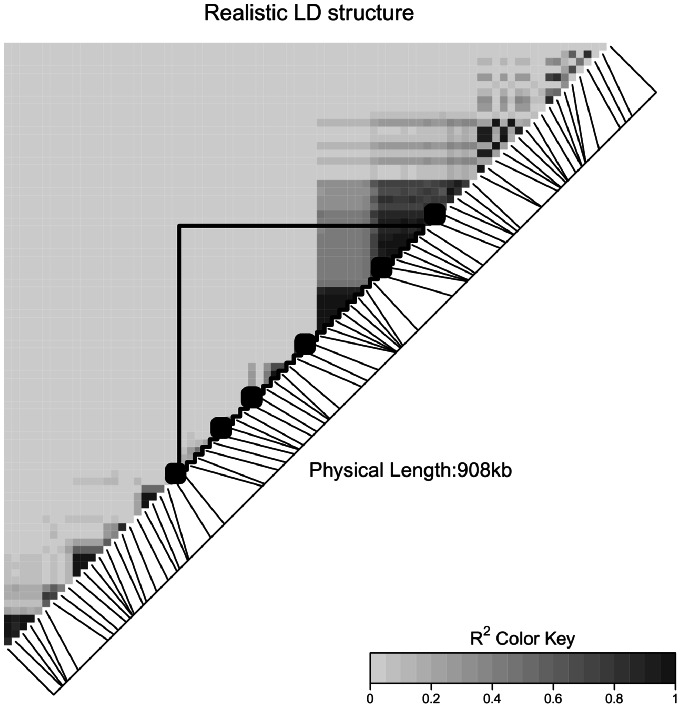
Heatmap of LD structure used in realistic data analysis. Figure 1 shows a heatmap illustrating the complex LD structure utilized in the realistic data analysis. The ∼900 kb region includes an approximately 400 kb “gene” flanked by approximately 250 kb of inter-genic space on each side. There are a total of 80 common SNPs (MAF>0.05) in the region, denoted by line which indicate their location in the genome. The six causal SNPs in our analysis are indicated in bold. All other SNPs are non-causal. There is a large, moderate-strong LD block on the upper end of the gene, which crosses from inside the gene into the inter-genic space. Other LD is fairly localized to small blocks.

We also used the observed LD structure in a sample of real genotype data as a starting point for simulation. We started by inferring phased haplotypes and population haplotype frequencies in a ∼900 kb region using fastPHASE [17]. These population estimates were then used to generate random pairs of haplotypes – representing a population of genotypes. The simulated realistic genotype data represents 80 unique SNPs spanning MAFs from 0.06 to 0.50 (mean  = 0.33 sd  = 0.15). We arbitrarily let the (approximately) middle 400 kb represent a gene, with approximately 250 kb of intergenic space on either side of the gene. Figure 1 illustrates the start-stop positions of the gene, the locations of the measured genotypes in and nearby to the gene, and the LD structure (*r^2^*) in the genotype sample.

As shown in Figure 1, 33 SNPs are contained within the gene (intragenic) and 47 SNPs are in the regions outside of the gene (intergenic with 23 on one side and 24 on the other). Figure 1 also illustrates (in bold) the locations of 6 SNP loci (4 intragenic and 2 intergenic) denoted as “causal” in our simulation. Thus of the 33 intragenic SNPS, 29 are non-causal and 4 are causal, while of the 47 intergenic SNPs, 2 are causal and 45 are non-causal. For the realistic genotypes, we considered four different relative risks (1.25, 1.5, 2.0, and 3.0) and sample sizes of 1000 and 2000 evenly split between cases and controls, for a total of 8 simulation settings. As is described further in the results section, we applied window and LD approaches to include or exclude different combinations of causal and non-causal SNPs.

### Phenotype simulation for simulated genotypes

Disease status was simulated following a method similar to that of Li and Leal [7]. Specifically, each causal SNP in the set was assigned a separate disease prevalence, computed as *P(D_U_) = *0.10/*i*, to represent that SNP's unique contribution to overall disease prevalence, where *i* =  the number of causal SNPs. Overall disease prevalence, *P(D)*, is approximately 10% in all cases, since *(1−P(D_U_))^i^* is approximately equal to 0.10 for all *i* = 1,2,4,8.

The probability of disease given an observed genotype at each causal SNP can then be simulated using a Bernoulli random variable for each causal SNP, with probability *P*(*D_U_/G_j_)* (*G* is the genotype at the site of interest, and takes values of *G* = 0,1,2 representing the number of minor (risk) alleles) and where conditional disease probabilities are computed as: *P(D/G = 0) = P(D_U_)/[(1−m)^2^+2γm(1−m)+(2γ−1)m^2^]*, *P(D/G = 1) = * γ*P(D/G = 0)* and *P(D/G = 2) =  (2γ−1) P(D/G = 0)* and where *m* is the MAF at the site, and we assume an additive disease model with relative risk, γ. If any of the causal SNP sites yield a “disease” status using the Bernoulli random variable, the person is deemed diseased. As noted earlier, large sets of genotypes were simulated acting as a large population, case/control status is simulated as described above, and then the appropriate number of cases and controls are randomly chosen from this population of all genotypes.

### SNP-set tests

All simulated data was analyzed using five recently proposed SNP-set tests, namely GATES [4], two different versions of VEGAS [3], logistic regression using principal components [5], and traditional logistic regression [5]. In the following paragraphs we briefly describe the five methods.

### GATES

The GATES method [4] is an extension of a Simes approach to combining multiple single marker genotype-phenotype tests applied at each SNP. Briefly, the method computes a standard linear trend test of association for each SNP with the disease phenotype, yielding a p-value for each SNP in the set. LD between the genotypes of all SNPs in the set is computed based on the sample. The individual SNP p-values are then combined (summed) in a manner which appropriately controls for the correlation structure – namely, small p-values from multiple highly correlated SNPs count less than small p-values from multiple non-correlated SNPs.

### VEGAS (VEGAS-SUM and VEGAS-MAX)

The VEGAS procedure [3] also combines individual SNP-phenotype statistics from a standard linear trend test of association. Without linkage disequilibrium, the sum of these statistics has a null distribution of chi-square with *n* degrees of freedom, where *n* equals the number of SNPs in the SNP set. To account for linkage disequilibrium, the VEGAS procedure uses a Monte Carlo approach with simulations from a multivariate normal to estimate the null distribution. First, a vector of independent standard Normal random variables is generated and multiplied by the Cholesky decomposition matrix of the matrix of pairwise LD values. Each element of the vector is then squared. This process is repeated and the gene-based statistic is calculated for each vector generating a null distribution. Two different gene-based statistics are suggested: the sum statistic (VEGAS-SUM), which is the sum of the chi-square SNP statistics, and the max statistic (VEGAS-MAX), which is the maximum of the chi-square SNP statistics. The gene-based p-value is simply the proportion of times the simulated gene test statistic exceeds the observed gene test statistic.

### Logistic regression using Principal Components (LR-PC)

Gauderman et al. [5] proposed a modified logistic regression approach to SNP set testing. First, principal components analysis is applied to the matrix of genotypes represented by all SNPs in the set. The first X principal components are retained, where X is the minimum set of principal components that explains at least 80% of the variability in genotypes. Logistic regression is then used to regress the disease phenotype onto the minimal set of X principal components. The gene-based p-value is calculated from comparing this model to the null model using a likelihood ratio test.

### Logistic regression (LR)

Gauderman et al. [5] also considered logistic regression applied directly to the genotype matrix. Here, each SNP is its own explanatory variable in the logistic regression model and a gene-based p-value is computed using a likelihood ratio test.

### Real data

As part of our evaluation of the performance of different SNP-set methods, we also extended a previous analysis by our group to an analysis of heart disease causing SNPs in the Framingham heart study sample. Details of the sample, genotyping technology and gene assignments are provided elsewhere [18]. We focused our analysis on a gene (VSTM4) on chromosome 10 which showed modest evidence of association in single marker analyses. The full dataset is currently available viadbGaP (http://www.ncbi.nlm.nih.gov/gap).

### HapMap data

In order to discuss the practical implications of our simulation analyses in situations where genome-wide application of SNP-set tests will occur, we explored SNP data as provided by HapMap. Specifically, we considered the Phase 3 CEU HapMap (HapMap 3 draft release #2, NCBI B36) sample, representing individuals of northern and western European ancestry [19]. SNPs were then classified as being intragenic (located within a protein-encoding gene; 577,306 SNPs) or intergenic (located in an intergenic region within 200 kb of a protein encoding gene; 488,942 SNPs) based on gene locations identified by Ensembl [20]. LD data on all HapMap SNPs is provided by HapMap for SNPs within 200 kb of each other.

## Results

### Changes in power when no LD is present

We first consider the 288 simulation settings for which there is no LD between SNPs. We summarized the general impact of simulation parameters on power through the use of a multiple regression model predicting power by each of the 6 simulation parameters as main effects (relative risk, number of causal SNPs, number of non-causal SNPs, MAF of causal SNPs, MAF of non-causal SNPs, and sample size). A separate model was fit for each of the five gene-based testing methods. Main effects models yielded *r^2^* values between 63% and 66%, suggesting that the main effects terms capture much of the observed variation in power values.

As expected, for all five tests, as the relative risk, sample size, or MAF of causal SNPs increased, the power of all tests significantly increased with similar estimated regression coefficients across the five methods (details not shown). The MAF of non-causal SNPs was not significantly related to power for any test except LR-PC, where power decreased as the MAF of non-causal SNPs increased. Changes in power across the other two simulation settings, the number of causal and non-causal SNPs, are the focus of our analysis here. First, for all five tests, as the number of non-causal SNPs increased, power decreased; while power increased with the addition of causal SNPs to the analysis. For all of our simulation settings, and averaged across the five SNP test sets, power declined by an average absolute amount of 0.0026 for each additional non-causal SNP included in the test, but can be as high as 0.0095. Table 1 indicates the average values for each of the five tests; little variation between tests was observed. The ratio of power gain for causal SNPs to power loss for non-causal SNPs provides a rough estimate of the number of non-causal SNPs that “cancel out” the effect of a single causal variant. As shown in Table 1, these ratios range from 6.1 to 11.5 for the five tests.

**Table 1:** Relationship of Power to Independent Non-Causal and Causal SNPs^1,2^.

Gene-Based Test	Estimated absolute power loss for one additional non-causal SNP#	Estimated absolute power gain for one additional causal SNP	Estimated number of non-causal SNPs which cancel out the power gained from one causal SNP
GATES	0.0025	0.0160	6.3
VEGAS-SUM	0.0028	0.0299	10.6
VEGAS-MAX	0.0025	0.0151	6.1
LR-PC	0.0023	0.0267	11.5
LR	0.0027	0.0305	11.4

1 Using a multiple regression model including all parameter and simulation results.

2 Assuming all other parameters are held constant.

### Changes in power when LD is present between non-causal SNPs

Next, we considered the impact of LD between the non-causal SNPs. The LD simulation parameters we added to the model were amount of LD (*r* = 0.5 or *r* = 0.9) and the number of LD blocks within the non-causal SNPs (1 or 2). Regression models similar to those described in the previous section were used to assess overall trends in power as related to the simulation parameters. Overall, the eight main effects terms explained most of the observed variability in power (model *r^2^* values ranged from 44% to 67%). The six main effects described in the previous section behaved the same in all cases (detailed results not shown), and so we focus our attention only on the two LD parameters.

For all five tests, power decreased very little when moving from one to two LD blocks; the main effect term in each of the regression models was non-significant. On the other hand, the amount of LD observed (high or low) was significantly related to the observed power of the test in three of the five models, though the direction of effect was different for different tests. For the GATES test, high LD between non-causal SNPs yielded significantly more power than low LD. A similar trend was observed for LR, though it was not statistically significant. For both VEGAS-max and VEGAS-sum increased LD was associated with significantly lower power. The LR-PC test performed very poorly in situations with high-LD and a large number of non-causal SNPs. Further investigation found that this approach was eliminating the causal variants from the analysis since principal components on the genotype matrix found more than 80% of the correlation in genotypes explained by non-causal variants alone. Figures S1–S5 illustrate the typical behavior of these tests. Importantly, these trends mean that the GATES test is more robust to the inclusion of non-causal variants, when those variants are in LD, while the VEGAS tests are less robust to the presence of non-causal variants in the presence of non-causal LD.

### Power change in the presence of LD between causal and non-causal SNPs

Next, we considered the impact of LD between causal and non-causal SNPs. Regression models similar to those described in previous sections were used to assess overall trends in power in relation to the simulation parameters. Overall, the seven main effects terms explained most of the observed variability in power (model *r^2^* values ranged from 64% to 87%). Most simulation parameters described earlier (sample size, RR, MAF, number of causal SNPs) behaved similarly here, so we focus our discussion only on the amount of LD and the number of non-causal SNPs.

When analyzing all SNPs simultaneously (causal and non-causal), the addition of non-causal SNPs was not related to changes in power for four of the five methods. The lone exception was VEGAS-max which yielded lower power with larger numbers of non-causal SNPs. We note that in all simulation settings considered in this analysis, all non-causal SNPs are in LD with a causal SNP. As seen earlier when investigating the relationship between power and amount of LD, different methods yielded different results. In this case, three of the five methods (GATES, VEGAS-sum and LR) showed significant power gain with higher levels of LD, while VEGAS- max showed significant power loss with higher levels of LD, while LR-PC showed an insignificant change in power.

A follow-up analysis which considered only non-causal SNPs in order to illustrate a situation where causal SNPs were not genotyped (e.g., if only using tagSNPs) showed similar patterns of association in almost all cases. The two exceptions were that the VEGAS-max test no longer showed significant power loss as LD increased, and the LR-PC test showed significant power gain as LD increased.

### Power change in a realistic LD situation

We now focus on how power changes for the different tests in a realistic LD situation (as depicted Figure 1). The focus of our analysis will be to evaluate the performance of the window and LD approaches for this data (see Introduction for details). As we have shown, the inclusion of non-causal SNPs can create significant power loss in many situations. Notably, within the “gene” there are 29 non-causal SNPs and 4 causal SNPs. Thus, even without adding any intergenic SNPs there are a large number of non-causal SNPs that will decrease power for the resulting tests of association. At window sizes of 50 kb, 100 kb, 150 kb and 250 kb, there were 3, 12, 29 and 35 non-causal SNPs added to the test, respectively. Note that there are two causal SNPs nearby to the gene, but outside of the gene boundaries (one upstream, and one downstream of the gene).

**Figure 2:**
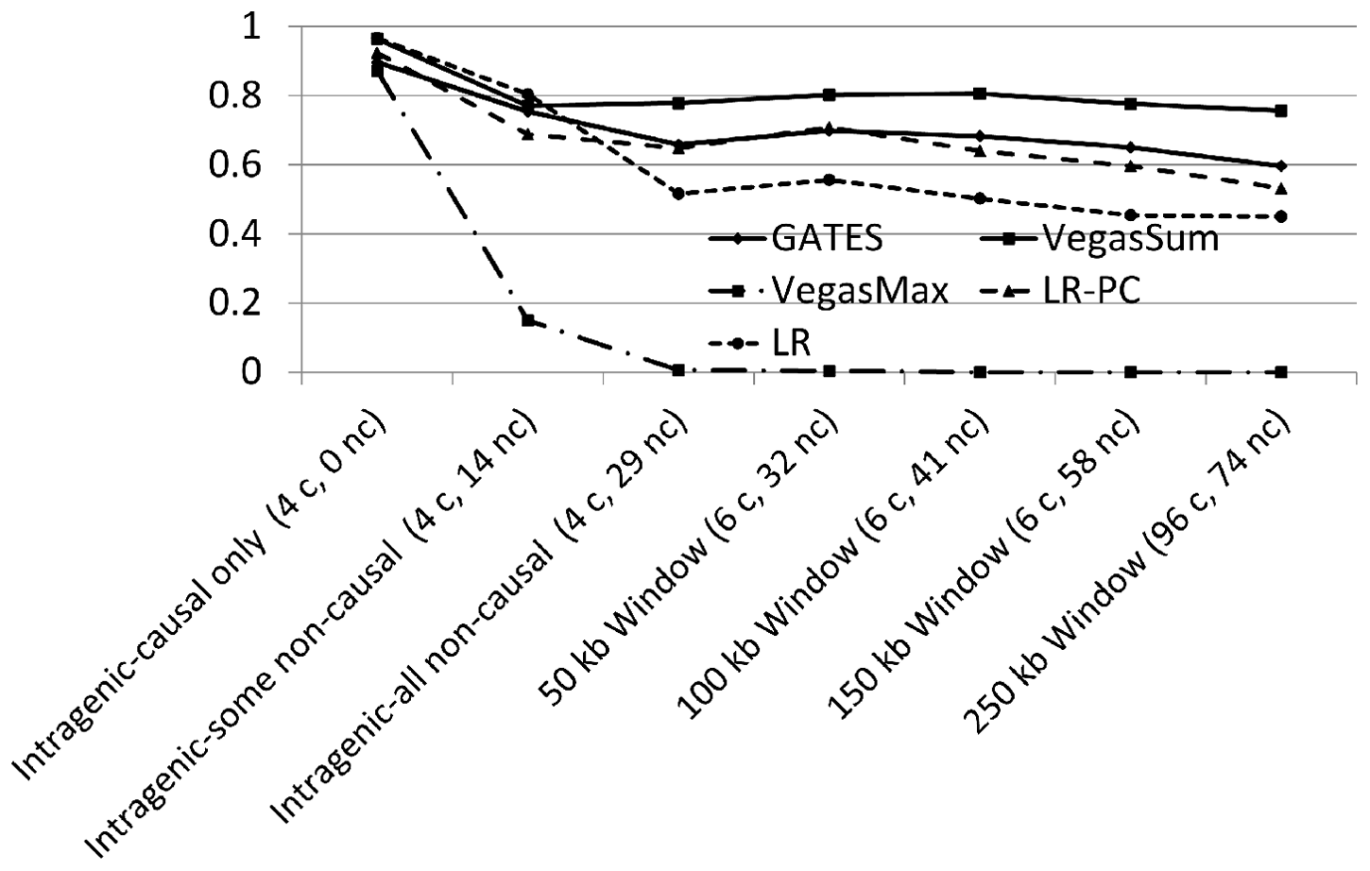
Power loss from the inclusion of non-causal SNPs with complex LD structure using a window approach to include inter-genic SNPs. Figure 2 illustrates the power loss observed for all five tests in the realistic LD structure simulation, as more and more non-causal SNPs are added to the test. In particular, a notable decline in power is noted for all tests as the number of intra-genic and inter-genic non-causal SNPs increases. The notable exception is when a small increase in power is observed for most tests when a small region (window) outside of the gene is included since two causal SNPs are located nearby to the gene. Power loss from the inclusion of non-causal SNPs can be substantial, in particular the inclusion of intra-genic non-causal SNPs substantially decreases power. Thus, using increasingly large windows only decreases power when no causal SNPs are located far from the gene.

Figure 2 illustrates similar trends to those seen earlier, namely as the number of non-causal SNPs increases, power decreases. As noted in the previous paragraph, as the window size increases, the number of non-causal SNPs included in the analysis also increases – decreasing power. We also illustrate two analyses where we did not include some or all intragenic non-causal SNPs in the analysis (all 29 dropped, or 14 of 29 dropped) to illustrate how better prior knowledge about which SNPs are causal can improve power. While Figure 2 illustrates only a specific simulation setting (prevalence  = 10%, Risk  = 2, Sample size  = 1000), other simulation settings showed results (detailed results not shown). In particular, aside from VEGAS-MAX which performed poorly in the presence of strong LD, all tests showed decreasing power from the inclusion of non-causal SNPs, and increased power from the inclusion of causal SNPs (e.g., the “bump” seen when moving from 4 to 6 causal SNPs).

**Figure 3:**
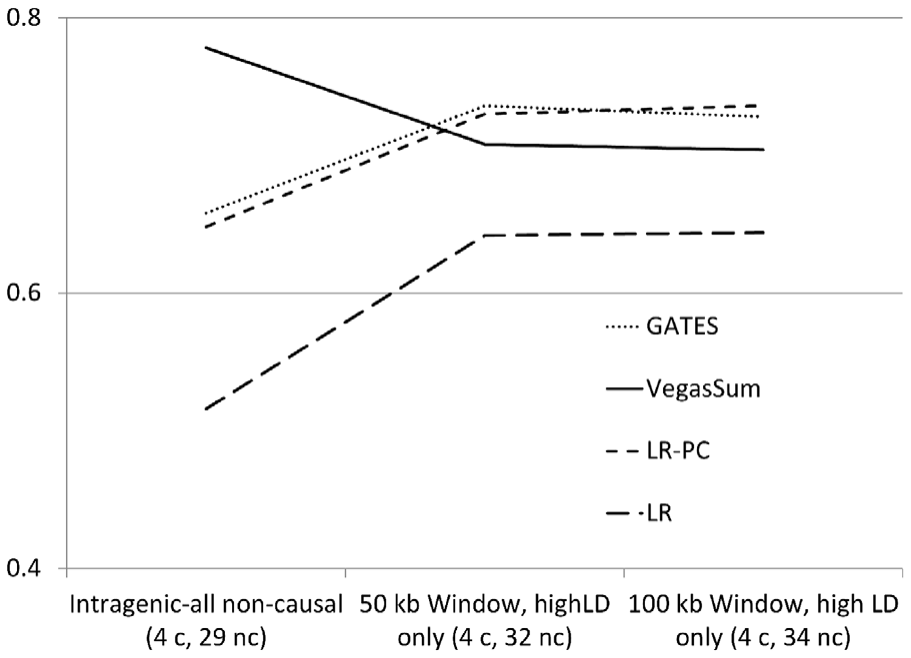
Power loss from the inclusion of non-causal SNPs with complex LD structure using an LD approach to include inter-genic SNPs. Figure 3 illustrates behavior observed for different methods when using an LD aware approach to assigning inter-genic SNPs to genes. In particular, an LD-aware approach limits the number of inter-genic SNPs assigned to the set, which reduces the number of non-causal SNPs assigned to the set if no causal SNPs are located outside of the gene. While GATES, LR and LR-PC showed improved power from the LD-aware approach, VEGAS-sum illustrated a loss in power from the addition of non-causal SNPs in LD with causal intra-genic SNPs.

We also considered the inclusion of intergenic regions around the SNP using a combined window-LD thresholding approach. In particular, only SNPs within a given window of the gene that exhibited LD of at least *r^2^*>0.7 with at least one SNP in the gene were included in the analysis. Figure 3 illustrates the results of this analysis for a particular simulation setting (similar results were seen for other settings). In particular, note that for three of the four tests (GATES, LR, and LR-PC) power increases with this approach. Power increases occur because the inclusion of the intergenic non-causal SNPs yield a stronger signal due to LD with intergenic causal SNPs. For VEGAS-sum, the power decreased because, as noted earlier, adding non-causal variants in LD tends to decrease power for VEGAS-sum. Results for VEGAS-max are not shown due to its poor performance.

### Performance of methods on real data

**Figure 4:**
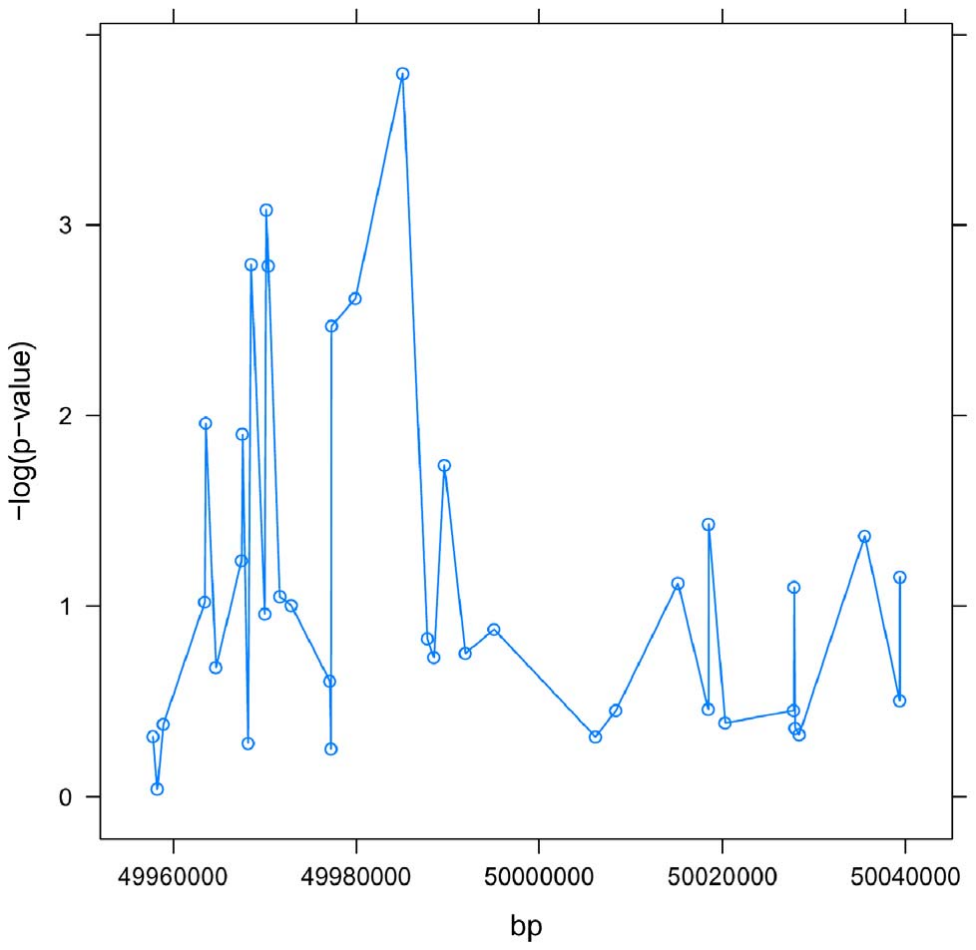
Manhattan plot for region in and around VSTM4. Figure 4 illustrates the –log_10_ tranformed p-values located within 50 kb of VSTM4. We see two distinct regions containing small p-values: (a) a region near 49,970,000 shows 3 SNPs with transformed p-values greater than 2.3 (p-value<0.005), and another region between 49,998,000 and 49,999,000 (overlapping VSTM4) also has three SNPs with transformed p-values greater than 2.3.

We applied GATES, VEGAS-SUM, VEGAS-MAX, LR-PC and LR to sets of SNPs in and around VSTM4, a 13 kb gene located at approximately 49.9Mb on chromosome 10. Figure 4 shows two distinct regions containing SNPs with small p-values. Table 2 shows the results of the five different SNP-set methods applied to the 5 SNPs within VSTM4, as well as to sets of SNPs using windows of increasing size. The VEGAS-MAX and LR methods yield relatively large p-values across the different sets, as compared to the other methods. The LR-PC and GATES methods both show the strongest association for the 5 SNPs within VSTM4, with slowly increasing p-values as more and more SNPs are added to the set. The VEGAS-SUM statistic shows the strongest evidence for association of the set when SNPs within 15kb are included – which includes all 6 SNPs showing the strongest Table 3 illustrates the LD structure between the 6 most significant SNPs and suggests some LD between the two regions. We note that none of the 38 individual SNP p-values would yield evidence of association strong enough to be considered genome-wide significant (p<1×10^−8^), and only two would be significant at a Bonferroni corrected significance level for the 38 SNPs in this region (0.05/38 = 0.0013; rs12245255 and rs4240498). The set methods (especially VEGAS-SUM) provide robust evidence of association in this region.

**Table 2:** P-values from individual and SNP-set approaches for the VSTM4 gene.

Region (SNP-set)	Total Number of SNPs	Additional SNPs with p-value less than 0.002 (p-value)^1^	GATES	VEGAS-SUM	VEGAS-MAX	LR-PC	LR
VSTM4	5	rs12245255 (0.00016)	0.0006	0.0034	0.011	0.0021	0.02
VSTM4+/−5kb	6	rs4298825 (0.003) rs4488117 (0.002)	0.0011	0.0023	0.035	0.0046	0.14
VSTM4+/−10kb	10	rs6537494 (0.0016)	0.0014	0.0014	0.060	0.0047	0.20
VSTM4+/−15kb	15	rs4240498 (0.0008) rs7074818 (0.0016)	0.0020	0.0007	0.16	0.013	0.22
VSTM4+/−25kb	25	none	0.0030	0.0010	0.24	0.015	0.12
VSTM4+/−50kb	38	none	0.0034	0.0024	0.33	0.041	0.003

1. To find the total number of significant SNPs in each SNP-set include all significant SNPs located in and above the row of interest.

**Table 3:** LD (r^2^) between most 6 associated SNPs (p<0.005 from individual marker tests).

Location (bp dow nstream of VSTM4)	SNPID	rs7074818	rs4240498	rs6537494	rs4298825	rs4488117	rs12245255
11696	rs7074818	1.00	0.90	0.94	0.37	0.38	0.84
10038	rs4240498	0.90	1.00	0.96	0.37	0.40	0.90
9824	rs6537494	0.94	0.96	1.00	0.40	0.41	0.90
2915	rs4298825	0.37	0.37	0.40	1.00	0.94	0.42
312	rs4488117	0.38	0.40	0.41	0.94	1.00	0.45
Intragenic	rs12245255	0.84	0.90	0.90	0.42	0.45	1.00

### Consideration of real LD structure

**Figure 5:**
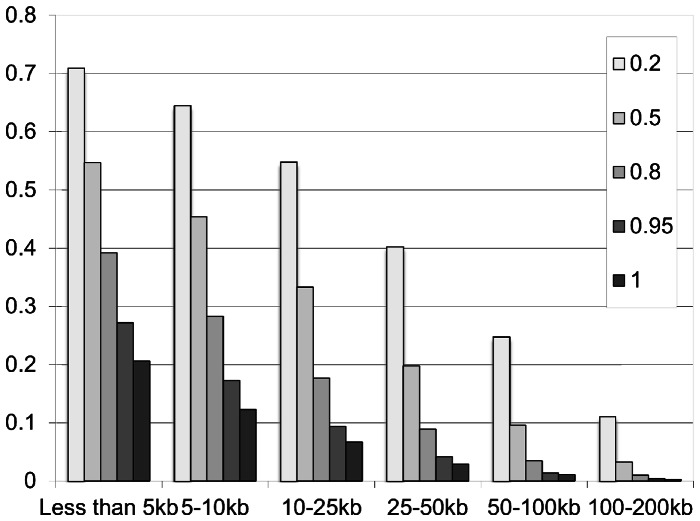
Relationship of LD block size and structure to window size from HapMap data. Figure 5 illustrates that as distance from the gene increases, the likelihood that a SNP is in LD with an intra-genic SNP decreases. Importantly, the likelihood of a SNP being in moderate LD (r^2^>0.70) more than10 kb from the boundary of the gene is only approximately 35%, with the likelihood of being in high LD even lower.

To provide a genome-wide view of the LD structure as it pertains to gene-based tests of association, we analyzed the LD structure of HapMap data. Figure 5 illustrates how LD structure relates to window size. As expected, blocks of high LD are of limited size, and so as distance from the end of the gene increases, the likelihood of an intergenic SNP being in LD with a SNP in the gene, declines dramatically.

## Discussion

Gene-based tests are being applied with increasing frequency to common SNPs (MAF>5%) directly measured by SNP microarrays or imputed in GWAS as an alternative to single-marker tests. Despite the promise that aggregating the signal from multiple causal variants will improve power and reduce multiple testing penalties, these methods have generally performed poorly in practice. In our analysis we investigated a variety of realistic factors potentially associated with power across two major classes of gene-based tests. First, we confirmed that all gene-based tests considered here illustrate well-known and expected relationships between power and sample size, relative risk, MAF and number of causal variants. Furthermore, we found that the inclusion of non-causal variants was detrimental to power for all methods. In fact, on average, it only took 6–12 independent non-causal variants to “cancel out” the effect of a single causal variant. This implies that unless more than 10–15% of all independent variants are causal, gene-based tests of common variants may be relatively ineffective. Complex LD structure, the differing behavior of different statistical methods to that LD structure, and variations in the impact of relative risk/MAF/sample size means that we should be hesitant to generalize that result to all situations. However, the fact remains that non-causal SNPs are substantially impacting power of gene-based tests.

The impact of non-causal SNPs is compounded when we consider that many investigators include inter-genic SNPs in gene-based analyses. If no causal SNPs are present in the inter-genic space, then researchers should only include inter-genic SNPs that are in LD with SNPs inside the gene – and, in this case, it is only beneficial for certain methods (e.g., GATES, LR), while this approach appears detrimental to other methods (e.g., VEGAS). As more and more genomic information becomes available, utilizing LD information in gene-based tests is becoming more practical than ever.

Window-based approaches are only reasonable when causal SNPs are in the inter-genic space. Of course, *a priori*, researchers do not know if causal SNPs are being included. However, increasingly, bioinformatics databases include annotations of inter-genic spaces (e.g., locations of regulatory elements) that can be used intelligently when adding inter-genic SNPs. Blind use of the window approach can no longer be recommended as best practice. But, if window-based approaches are to be used to capture LD structure, only very small windows should be used since, as shown in Figure 4, a very small percentage of SNPs beyond 10 kb are in moderate to high LD with SNPs in the gene. We acknowledge that this approach will ignore regulatory elements located more than 10 kb from the gene. Further analysis is needed to explore distributions of gene sizes, SNP distributions, and regulatory elements in order to further refine these general guidelines.

However, perhaps even more importantly than SNPs in the inter-genic space, is the impact that better prioritization of intragenic SNPs will have on power. For example, as we anticipate more and more sequence data available, we can anticipate that (a) all causal SNPs will be typed and (b) that predicted functional impact of SNPs can be integrated into the analysis. For example, given exonic sequence data, it may be practical to include only non-synonymous inter-genic SNPs in the analysis, thus increasing the causal to non-causal SNP ratio and, potentially, improving statistical power. Additionally, if all SNPs are typed (directly sequenced or imputed), then we longer will need to rely on tag SNPs (non-causal SNPs in LD with causal SNPs) to capture the causal signal. Further consideration is needed to explore how gene-based tests should be applied to common variants when investigating sequence data.

Recently, given the advent of next-generation sequencing data, gene-based testing methods which incorporate both common and rare variants have been proposed. Further work is needed to see how the conclusions found here apply to those methods. However, the effect of non-causal variants is likely the same since methods which focus only on rare variants have been shown to suffer power loss in the presence of non-causal variants (e.g., Li and Leal 2008). Ultimately, methods are needed which are more robust to the inclusion of non-causal variants. A promising approach has recently been proposed by Liu et al. (unpublished manuscript).

As more knowledge of “typical” genetic architectures becomes available, more sophisticated analyses comparing single and multiple marker methods will be possible that can explicitly consider the tradeoff of multiple testing penalties for power in the presence of differing numbers of causal variants, their relative risks and allele frequency distribution, as well as the impact of non-causal variants.

Lastly, our analysis considers only five of a very large, and growing, set of gene-based tests. Notably, we only considered self-contained tests and did not consider competitive tests in our analysis. We use the GATES/VEGAS tests as representatives of tests that combine single marker p-values and use LD structure to account for correlation between genotypes. LR and LR-PC were selected as representatives of gene-based tests requiring the full genotype-phenotype matrix and use regression or regression-like approaches to assess significance of a set of markers. Given the disparate relationships between LD structure and power, even between the methods selected here mean that some caution is needed when projecting our conclusions beyond these methods.

Our analysis suggests that one reason for the poor performance of gene-based tests of association for common variants is due to limited power in the presence of a large percentage of non-causal variants. This finding suggests that window-based methods of assigning SNPs to genes should not be used, especially in light of increasing knowledge of the human genome. Methods are needed which are more robust to the inclusion of non-causal variants, though better a priori prediction of causal variants using bioinformatics methods will also substantially improve power.

## Supporting Information

Figures S1–S5 **Power loss from the inclusion of non-causal SNPs with LD between non-causal SNPs.** Figures S1–S5 illustrate power loss for the GATES, VEGAS-SUM, VEGAS-MAX, LR and LR-PC tests, respectively, due to the inclusion of non-causal SNPs for four combinations of LD blocks (1 or 2) and low or high LD (r = 0.5 or r = 0.9). Other simulation settings include: four causal SNPs, a combined relative risk of 2.00, a total sample size of 4000 individuals, and a MAF of 30% for all SNPs.
(PDF)
