# Supplementary figures and images for: Assessing Methods for Assigning SNPs to Genes in Gene-Based Tests of Association Using Common Variants
Source: PLoS One. 2013 May 31;8(5):e62161. doi: 10.1371/journal.pone.0062161 (PMC3669368; doi:10.1371/journal.pone.0062161)

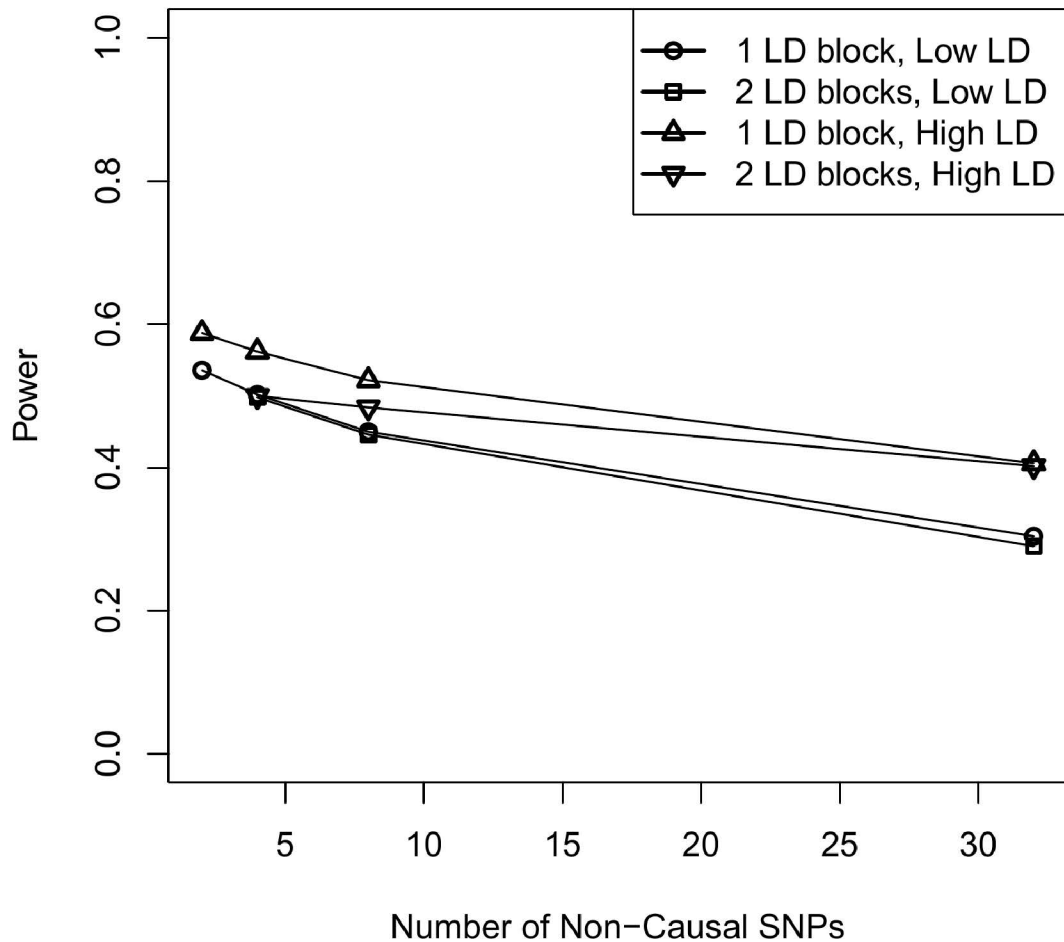

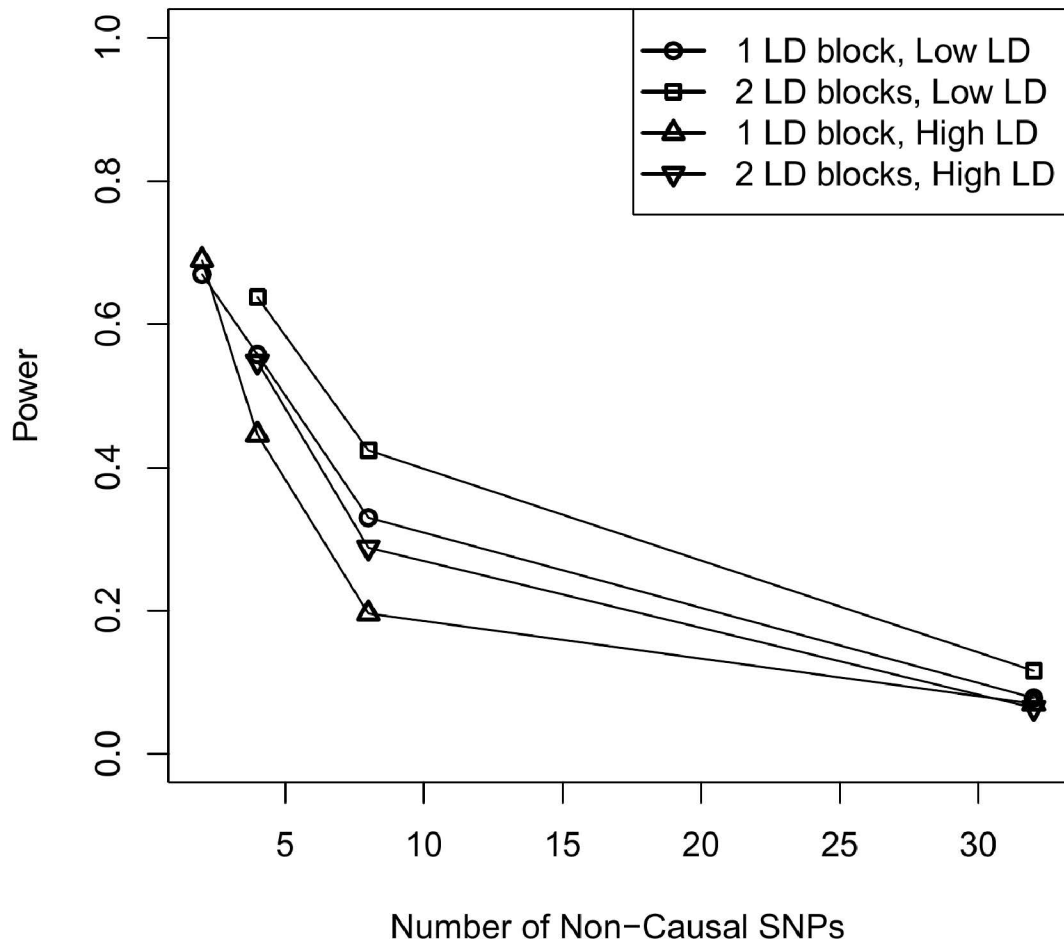

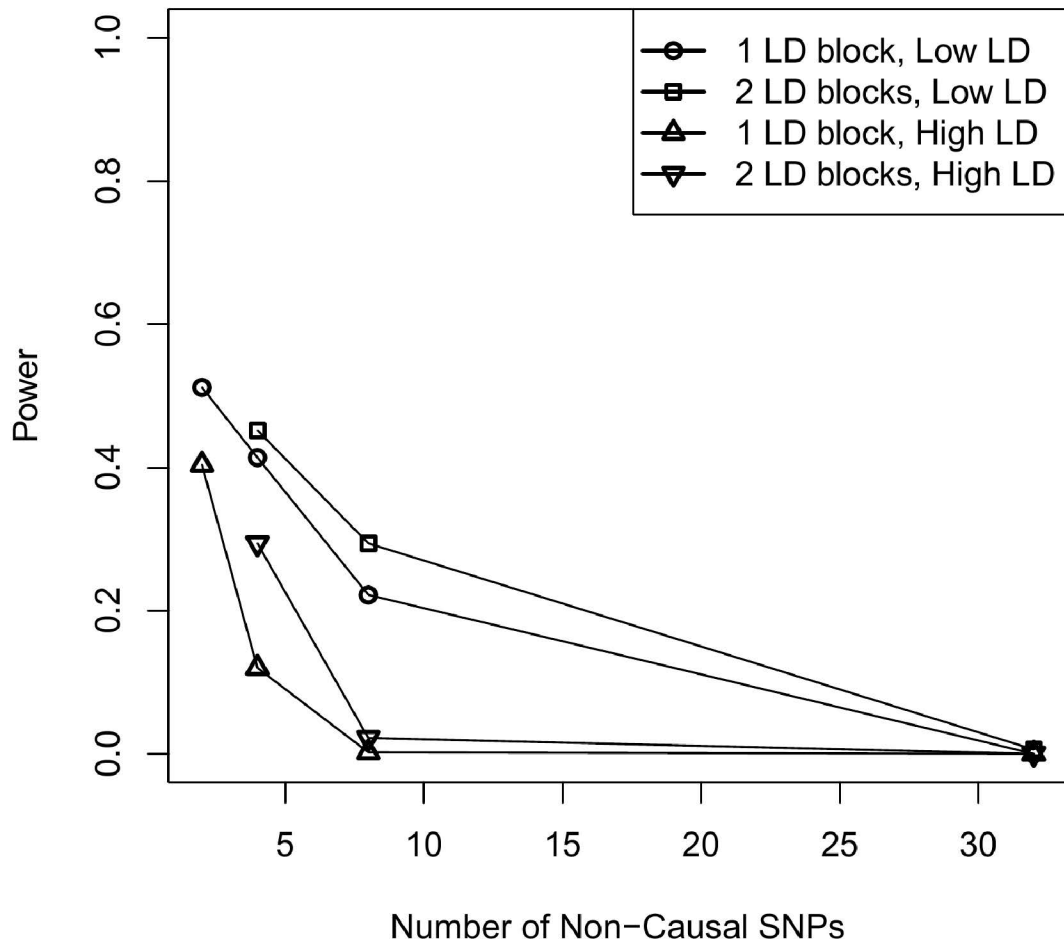

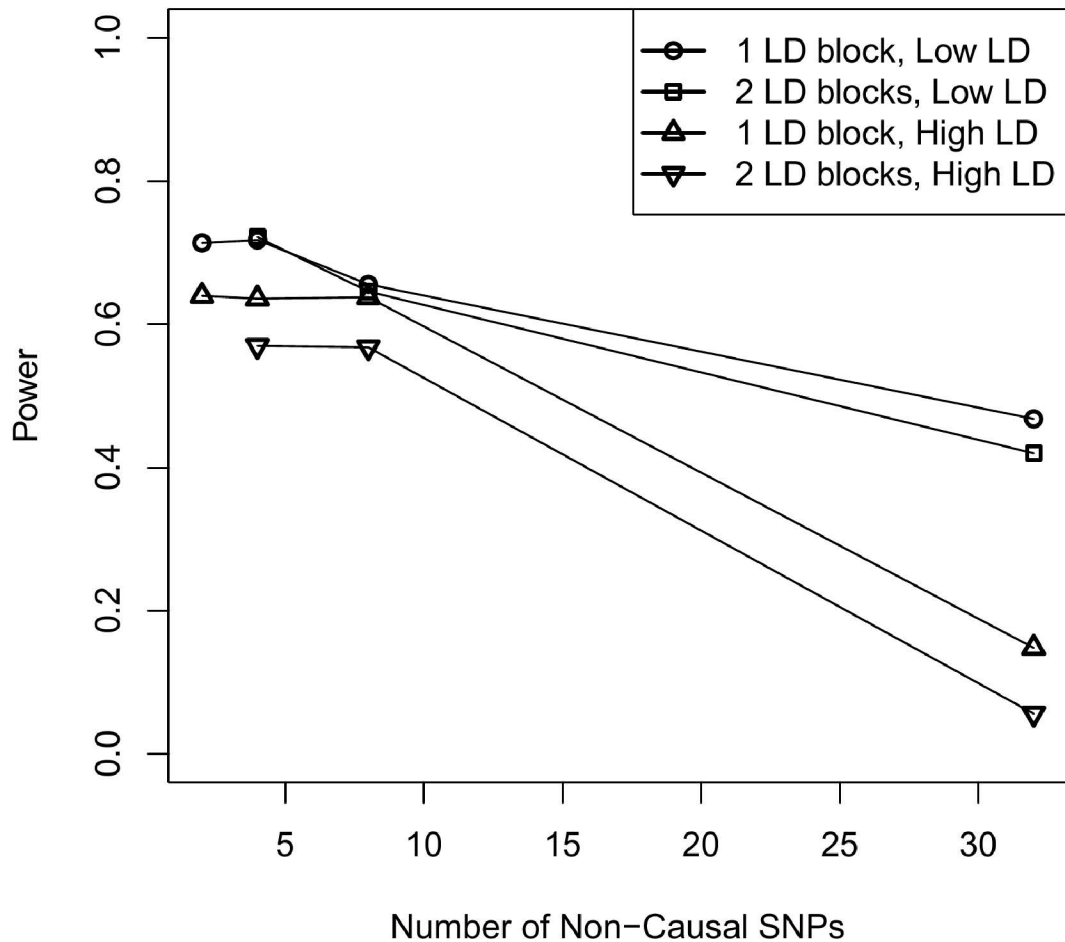

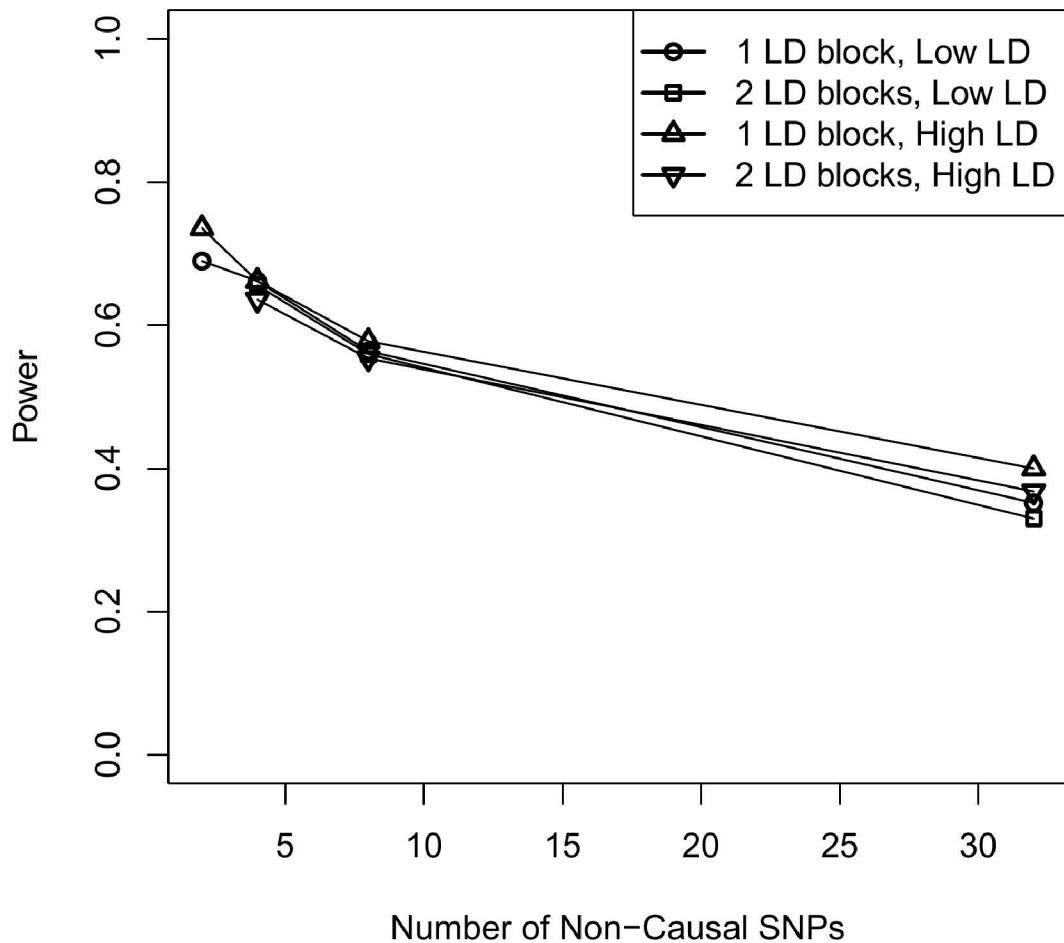

Supplement: Figures S1–S5 — Power loss from the inclusion of non-causal SNPs with LD between non-causal SNPs. Figures S1–S5 illustrate power loss for the GATES, VEGAS-SUM, VEGAS-MAX, LR and LR-PC tests, respectively, due to the inclusion of non-causal SNPs for four combinations of LD blocks (1 or 2) and low or high LD (r = 0.5 or r = 0.9). Other simulation settings include: four causal SNPs, a combined relative risk of 2.00, a total sample size of 4000 individuals, and a MAF of 30% for all SNPs. (PDF) [file pone.0062161.s001.pdf]
